# Explanation and inference: mechanistic and functional explanations guide property generalization

**DOI:** 10.3389/fnhum.2014.00700

**Published:** 2014-09-11

**Authors:** Tania Lombrozo, Nicholas Z. Gwynne

**Affiliations:** Department of Psychology, University of California, BerkeleyBerkeley, CA, USA

**Keywords:** explanation, teleological explanation, functional explanation, category-based induction, inference, property generalization, induction, causal reasoning

## Abstract

The ability to generalize from the known to the unknown is central to learning and inference. Two experiments explore the relationship between how a property is explained and how that property is generalized to novel species and artifacts. The experiments contrast the consequences of explaining a property mechanistically, by appeal to parts and processes, with the consequences of explaining the property functionally, by appeal to functions and goals. The findings suggest that properties that are explained functionally are more likely to be generalized on the basis of shared functions, with a weaker relationship between mechanistic explanations and generalization on the basis of shared parts and processes. The influence of explanation type on generalization holds even though all participants are provided with the same mechanistic and functional information, and whether an explanation type is freely generated (Experiment 1), experimentally provided (Experiment 2), or experimentally induced (Experiment 2). The experiments also demonstrate that explanations and generalizations of a particular type (mechanistic or functional) can be experimentally induced by providing sample explanations of that type, with a comparable effect when the sample explanations come from the same domain or from a different domains. These results suggest that explanations serve as a guide to generalization, and contribute to a growing body of work supporting the value of distinguishing mechanistic and functional explanations.

## Introduction

Suppose you learn that a particular species of mushroom contains a fatal toxin. Which other species of mushroom are likely to contain fatal toxins? One strategy for generalizing from known to novel cases is to consider *why* a property holds in the known case, and to determine whether those reasons extend to novel cases. For example, if you explain the mushroom's toxin as the product of a particular metabolic process, you might be inclined to judge other mushrooms sharing that metabolic process as similarly poisonous. If you instead explain the toxin as a biological adaptation to deter fungivores, you might be inclined to judge other mushrooms facing similar predators as poisonous.

In this paper we explore the hypothesis that explanations can guide the generalization of properties from known to novel cases. Explanations typically relate what's being explained (the explanandum) to more general patterns or regularities (Wellman and Liu, 2007; Williams and Lombrozo, 2010, 2013; Lombrozo, 2012), and in so doing highlight aspects of the explanandum that are likely to generalize to new cases (Lombrozo and Carey, 2006). For example, in explaining a mushroom's toxin by appeal to local predators, one (implicitly) invokes a specific relationship between that toxin and those predators, and also more general relationships between predation and defense, or even between ecological conditions and biological traits more generally. These explanatory patterns can inform how a property is generalized by influencing which prior beliefs are consulted, and which aspects of the known and novel cases are deemed relevant to whether a given property of the first applies to the second.

At a broad level, different *kinds* of explanations could correspond to different higher-order generalizations, or abstract “templates” for generalizations of different kinds. Here we consider *mechanistic* explanations, which explain by appeal to parts or processes, and *functional* explanations, which explain by appeal to functions or goals. Explaining a mushroom's toxin as the product of a particular metabolic process would thus qualify as mechanistic, while explaining the toxin as a biological adaptation to deter fungivores would qualify as functional. As we elaborate below, some scholars have proposed that mechanistic and functional explanations reflect different “stances” or “modes of construal” (Dennett, 1989; Keil, 1995), which makes them especially promising candidates as explanations that pick out different ways in which one might think about, and subsequently generalize, aspects of the explanandum.

In the two experiments that follow, we present participants with novel biological organisms or artifacts, along with information about the proximate causes and functions of one of its features. We then solicit, provide, or prime an explanation of a particular type (mechanistic and/or functional) and examine subsequent patterns of property generalization to other items with that feature. Using this basic task, we can investigate the extent to which different kinds of explanations guide generalization, as well as several related issues. These related issues include the role of explanations vs. general causal knowledge in guiding generalization, whether different bases for generalization compete, and whether explanatory modes can be primed within and across domains. In the remainder of the introduction, we explain these issues in greater detail.

### Explanation and property generalization

Beginning with the influential work of Carey (1985) and Osherson et al. (1990), cognitive scientists have studied property generalization as a window into inductive reasoning. A consistent finding is that property generalization is influenced by the similarity of the categories involved. For example, if porcini mushrooms are judged more similar to chanterelles than to shitakes, a property of porcini mushrooms should be extended to chanterelles over shitakes. However, the *property* being generalized influences *which* similarity relationships are relevant. This is nicely demonstrated in a study by Heit and Rubinstein (1994), in which participants were presented with a generalization task involving animals that were either anatomically similar–such as whales and bears–or behaviorally similar–such as whales and tuna. They found that the property being generalized determined whether anatomical or behavioral similarity was used to guide generalizations. When generalizing across anatomically similar animals (from bears to whales), an anatomical property (such as having a liver with two chambers that act as one) supported a stronger generalization than a behavioral property (such as traveling in a zig-zag trajectory). When generalizing across behaviorally similar animals (from tuna to whales), judgments followed the opposite pattern. Based on these findings, Heit and Rubinstein (1994) suggested that prior knowledge is “used dynamically to focus on certain features when similarity is evaluated” (p. 420).

Explaining *why* a category has a given property may be a mechanism by which prior beliefs are invoked to constrain the similarity relations guiding generalization (Lombrozo, 2006, 2012; Vasilyeva and Coley, 2013; Williams and Lombrozo, 2013). Support for this proposal comes from studies by Sloman (1994) and Rehder (2006). Sloman provided participants with an initial claim (such as: many secretaries “have a hard time financing a house” or “have bad backs”), and asked them to evaluate a related claim (such as: many furniture movers “have a hard time financing a house” or “have bad backs”). Critically, the properties involved were varied such that the provided and evaluated claims sometimes supported a common explanation (moderate income for trouble financing a house), and sometimes supported different explanations (a sedentary job vs. heavy lifting for back problems). Sloman found that claims were assigned a higher probability when they were conditioned on a statement with a common explanation than when they were conditioned on a statement with a competing explanation, suggesting that explanations guided generalization. Rehder (2006) employed a property generalization task similar to Sloman (1994), but generated novel categories with properties that participants would not have prior knowledge to explain. Rehder found that when participants were provided with an explanation for the property being generalized, they generalized the property to new items that shared that explanation, overriding effects of overall similarity. These findings suggest that explanations are spontaneously consulted in property generalization when participants possess relevant prior beliefs (Sloman, 1994), and inform judgments when provided (Rehder, 2006).

One way in which the present studies differ from this prior work is in considering cases for which multiple explanations for a property are concurrently true, such as the mechanistic and functional explanations for the mushroom's toxin offered above. Such cases can help us isolate the role of explanations, in particular, from causal beliefs more generally. To illustrate this point concretely, consider an example from Rehder (2006, Experiment 3). Some participants learned about “kehoe ants” along with several of their features, such as whether they possess thick blood or a slow digestive system. They were then asked whether properties of an original ant would hold of novel exemplars that shared few features or many features. Critically, some of the properties were presented with causal explanations that linked them to one of the features. For example, participants might learn that the ant “is immobile in cold weather,” and that “the immobility in cold weather is caused by the thick blood.” When provided with this explanation linking immobility to thick blood, participants generalized the property of immobility to other ants that also had thick blood, with very little influence of the number of additional features that were also shared (i.e., of global similarity). Thus, effects of explanation were demonstrated by comparing participants who received *different causal information*: some of them were told that immobility is caused by thick blood, others were not. In the present experiments, all participants receive the same information about causal relationships between features. As a result, the current experiments isolate effects of explanation that operate beyond the causal relationships those explanations presuppose.

### Mechanistic and functional explanations as distinct explanatory modes

Both philosophers and psychologists have suggested that the distinction between mechanistic and functional explanations is a deep one (Dennett, 1989; Keil, 1995; see also Aristotle, *Metaphysics*). For example, Keil argues that mechanistic and functional explanations reflect innate “modes of construal” (1995), while Dennett argues for the existence of distinct stances, among them a design stance featuring functional reasoning, and a physical stance involving more mechanical reasoning. Despite these intriguing suggestions, only a handful of studies have examined the cognitive bases and consequences of different kinds of explanation. These studies have found that functional and mechanistic explanations are both understood causally (Lombrozo and Carey, 2006), and that functional explanations are often preferred (Kelemen, 1999; Lombrozo et al., 2007; Kelemen and Rosset, 2009). In particular, Kelemen (1999) argues that children and adults are “promiscuously teleological,” finding functional explanations especially compelling because we evolved as social animals for whom intentional, goal-based reasoning was essential and pervasive.

Recent studies have also found that explanation type can influence performance on basic cognitive tasks, such as categorization (Lombrozo, 2009) and causal attribution (Lombrozo, 2010). To illustrate, consider Lombrozo (2009), in which participants learned about novel categories such as “holings,” a kind of flower with brom compounds in their stems, causing them to lean over as they grow, in turn allowing their pollen to be spread by field mice. Participants were asked why holings bend over, which could be explained mechanistically (by appeal to brom compounds) or functionally (by appeal to pollination). The kind of explanation participants provided predicted later judgments about category membership. Specifically, participants learned about two mystery flowers, one with brom compounds and the other with a bending stem, and were asked which was more likely to be a holing. Participants who provided a mechanistic explanation privileged causally prior features, replicating previous effects of “causal status” (Ahn, 1998), while participants who provided functional explanations did the reverse. These findings suggest that mechanistic and functional explanations have consequences for reasoning that could extend to property generalization. They also suggest that people can invoke explanatory knowledge flexibly to guide reasoning.

In the experiments that follow, we investigate two questions about the relationship between mechanistic and functional reasoning. First, we measure baseline preferences for providing one kind of explanation or another, as well as baseline tendencies to generalize according to shared mechanisms or shared functions. These preferences potentially bear on the idea that teleo-functional reasoning reflects a cognitive default (Kelemen, 1999) or that functional features can be especially diagnostic of category membership (Lombrozo and Rehder, 2012), on the one hand, or that (proximate) causes are more conceptually central than effects, on the other (Ahn, 1998; Cimpian and Markman, 2009). However, because it's not possible to generate explanations that are matched in all respects *except* for being mechanistic vs. functional, we caution against overly strong interpretations of any baseline preferences–instead, we think it's more meaningful to consider whether mechanistic and functional explanations are differentially predictive of corresponding patterns in generalization. To the extent that each explanation type reflects a “stance” or “mode of construal,” one might expect explanations of that type to predict corresponding generalizations. That is, we can investigate whether providing or receiving a mechanistic explanations is predictive of generalization on the basis of common proximate causes, and whether providing or receiving a functional explanation is predictive of generalization on the basis of common functions.

Second, we consider whether different bases for generalization are in competition. In other words, does generalizing on the basis of common proximate causes tend to preclude generalizing on the basis of common functions, and vice versa? If different stances or modes of construal compete with each other, one might expect greater reasoning of one type to predict reduced reasoning on the basis of the alternative. On the other hand, if both kinds of reasoning are reinforcing or can operate in parallel, one would not expect to see this kind of competition. Investigating a related question, Heussen (2010) found that functional explanations were discounted in favor of mechanistic explanations, but only for artifacts. This provides some evidence that explanations of different types can compete, but it remains an open question whether this kind of competition is more widespread, extending to generalization and to the biological domain.

### Domain differences in mechanistic and functional reasoning

While research on property generalization has tended to neglect domain as a factor that could influence judgments, a variety of researchers have suggested that mechanistic and functional explanations are more or less privileged in different domains. For example, Atran (1995) suggests that functional reasoning is native to a “living thing” module, while Kelemen (1999) suggests that teleo-functional reasoning is fundamentally linked to goal-directed action, but “promiscuously” extended to other domains. Keil (1995) suggests that functional reasoning is not innately tied to any particular domain, but is privileged within folk biology early in development. Finally, Lombrozo and Carey (2006) suggest that functional explanations are related to causal assumptions that crosscut domains. However, they also suggest that functional explanations will seem more appropriate when they fit a general and familiar schema, and some domains, such as folk biology, may furnish such schemas more readily than others. All of these views support a role for both mechanistic and functional explanations for biological organisms and for artifacts–the two domains explored here–but raise the possibility that the relative importance of the two explanation types could differ across domains and influence property generalization, or that the “type” of functional reasoning induced by functional explanations could differ across domains.

We thus explore two questions involving the role of domain in mechanistic and functional reasoning. First, we measure baseline preferences for different kinds of explanations and generalizations across domains. According to different proposals, functional reasoning is most natural for artifacts, for biological kinds, or is equivalent across these two domains. Again, however, we caution against a strong interpretation of baseline preferences. It's difficult to generalize beyond our stimulus materials: a handful of items is hardly representative of an entire domain, so “domain” differences could simply reflect the properties of our items. We therefore place greater weight on our second question, which concerns the extent to which “functional reasoning” is domain general vs. domain specific. Put differently, is functional reasoning about artifacts basically the same as functional reasoning about biological organisms? Or does each domain have a proprietary form of functional reasoning, such that cross-domain cases of functional reasoning are only weakly or analogically related? To ask these questions, we investigate–in Experiment 2–whether people can be primed to adopt a particular explanatory mode, and if so whether such priming is more effective (or only effective) *within* a given domain vs. a case that crosses domains.

### Overview of experiments

In sum, previous studies support the proposal that explanations inform property generalization, and that the difference between mechanistic and functional explanations is cognitively significant. However, previous research has not investigated whether explanations of different types predict subsequent generalization, how a property is generalized when more than one explanation is available, whether mechanistic and functional generalization are in competition, or whether explanatory modes can be experimentally induced within or across domains. To explore these issues, two experiments examined the relationship between the type of explanation offered for a property (mechanistic or functional) and how that property is generalized (according to common mechanisms or common functions) when the explanation type is freely generated (Experiment 1), experimentally provided (Experiment 2), or experimentally induced by a same-domain or cross-domain prime (Experiment 2).

## Experiment 1: spontaneous explanations predict patterns of generalization

In Experiment 1 participants learned about a novel category from the domain of biological kinds (e.g., a plant called a narp) or artifacts (e.g., a kind of garment called a draham). They were then asked to explain a feature of that category (e.g., a speckled pattern for narps; thick cloth for drahams), and to generalize properties of the feature (e.g., high contrast for the pattern on narps; a tight weave for the cloth used for drahams) to novel categories that shared common mechanisms or common functions.

The experiment tests the hypotheses that participants who explain features mechanistically will generalize properties to items with a shared mechanism more readily than those who do not provide mechanistic explanations, and that participants who explain features functionally will generalize properties to items with a shared function more readily than those who do not provide functional explanations. The experiment also allows us to investigate whether mechanistic and functional generalization compete, and to measure baseline preferences for explanation and generalization types across domains.

### Methods for experiment 1

#### Participants

Two-hundred-fifty-two workers on Amazon Mechanical Turk (155 male, 97 female; Age: *M* = 30, *SD* = 9) completed the task in exchange for monetary compensation. All participants provided informed consent, following a human subjects protocol approved by UC Berkeley's Institutional Review Board. Participation was restricted to workers with IP addresses within the United States, a HIT approval ratings of 95% or higher, and at least 50 previously-approved HITs. An additional 36 participants were excluded for leaving numerical responses or explanations blank (2) or for failing an instructional manipulation check (34) that was modeled on Oppenheimer et al. (2009) and presented after the main task; it required participants to read instructions closely in order to pass.

#### Materials and procedure

Participants learned about a novel biological kind or artifact with a feature that was generated by a proximate cause and that supported a function. Below is a sample item (see Table 1 for additional stimuli):

A narp is a kind of plant with a speckled pattern. Biologists have discovered that in narps, the speckled pattern is caused by the XP2 gene. Having a speckled pattern attracts butterflies, which play a role in pollination.

**Table 1:** **Stimulus materials for Experiments 1 and 2**.

**Domain**	**Category description**	**Generalized properties**
Biological organisms	A narp is a kind of plant with a speckled pattern. Biologists have discovered that in narps, the speckled pattern is caused by the XP2 gene. Having a speckled pattern attracts butterflies, which play a role in pollination.	Dense pattern
		High-contrast pattern
		Reddish pattern
	A slive is a kind of mammal with a furry tail. According to scientists, the fur on slives' tails develops as a result of exposure to UV light. Having a furry tail also serves an important function: it helps keep the tail warm.	Rough tail fur
		Dense tail fur
		Multi-colored tail fur
	A brollig is a kind of reptile with stripes. In brolligs, the stripes are caused by minerals in the reptile's diet. Having stripes also has an important function: it helps brolligs hide from predators.	Thin stripes
		Jagged stripes
		Dark-colored stripes
	A flivvet is a kind of bird with blue eyes. Biologists know that in flivvets, the eyecolor results from a pigment called the P7 pigment. Having blue eyes helps the birds absorb sunlight to produce essential vitamins.	Almost violet in color
		Very small
		Detect polarized light
Artifacts	A draham is a kind of garment made from thick cloth. The cloth is thick because it is woven on a special, double loom. The thickness serves an important function: it protects the wearer from rough underbrush.	Tight weave
		Multiple colors
		Heavy cloth
	A stranton is a kind of device with a translucent exterior. The translucence is caused by a compound called polycleristyrene. Having a translucent exterior is important, because it allows internal parts to be solar-powered.	Thin exterior
		Shiny exterior
		Scratch-resistant
	A blig is a kind of paintbrush with firm bristles. The bristles are firm because they are treated with a pigment called P7. Having firm bristles is important because bligs are used to paint inside fine cracks in wood.	Stretchy bristles
		Transparent bristles
		Thick bristles
	A zimb is a kind of lamp with a red LED inside. The LED is red because it is created with polyrensedis, a red dye. Having a red LED is important because it can then be used to attract a kind of firefly that responds to red light.	Pale color
		Yellowish from afar
		Larger than usual LED

Participants were then asked to explain why category members typically have the target property (e.g., “In a sentence, why do narps have a speckled pattern?”). This question is deliberately ambiguous between a request for a mechanistic explanation (“Because of the XP2 gene”) and a request for a functional explanation (“Because it attracts butterflies for pollination”); participants could provide either explanation, both explanations, or neither explanation.

Participants then learned about two additional novel artifacts or organisms, where one shared the same proximate cause and target feature as the original item, but had a different function, and the other shared the same target feature and function, but had a different proximate cause. The introduction of each new category was followed by three generalization judgments. Participants learned about a property of the target feature in the initial category, and were asked to determine the probability that the new categories likewise shared that property. Ratings were made on a 9-point scale, with the first point labeled “very unlikely,” the final point “very likely,” and the midpoint “neither likely nor unlikely.” Below are sample questions for the narp category:

Tomas are another kind of plant with a speckled pattern. The speckled pattern in tomas is caused by the XP2 gene, as it is in narps, but its function is different: it is to provide camouflage.

Suppose you find out that the speckled pattern of narps is very dense. How likely do you think it is that the speckled pattern of tomas is also very dense?

Suppose you find out that the speckled pattern of narps is very high in contrast. How likely do you think it is that the speckled pattern of tomas is also very high in contrast?

Suppose you find out that the speckled pattern of narps is reddish. How likely do you think it is that the speckled pattern of tomas is also reddish?

Laks are another kind of plant with a speckled pattern. The speckled pattern in laks is caused by a different gene, the YZL gene, but the speckled pattern has the function of attracting butterflies for pollination, as it does for narps.

Suppose you find out that the speckled pattern of narps is very dense. How likely do you think it is that the speckled pattern of laks is also very dense?

Suppose you find out that the speckled pattern of narps is very high in contrast. How likely do you think it is that the speckled pattern of laks is also very high in contrast?

Suppose you find out that the speckled pattern of narps is reddish. How likely do you think it is that the speckled pattern of laks is also reddish?

The two novel categories and their corresponding generalization questions were presented in a random order for each participant. Each participant completed the task for a single category, either biological or artifact, making *domain* a between-subjects factor with two levels. There were four possible base categories for each domain. Participants were randomly assigned to one of these possible categories.

### Results and discussion for experiment 1

We first report analyzes concerning generated explanations, then those concerning generalization judgments, and finally consider their relationship.

#### Generated explanations

Participants' explanations were coded for whether they included a mechanistic explanation and whether they included a functional explanation (see Table 2). An initial coder coded all explanations blind to condition; a second coder coded 25% blind to condition, yielding 100% agreement. Overall, participants provided mechanistic explanations more often for biological organisms (61 of 126 explanation) than for artifacts (30 of 126), χ^2^_(1, *N* = 252)_ = 16.53, *p* < 0.001, but the proportion of functional explanations did not differ significantly across domains: 102 of 126 explanations for artifacts vs. 91 of 126 for biological organisms, χ^2^_(1, *N* = 252)_ = 2.68, *p* = 0.102. In both domains, participants were more likely to provide functional explanations than mechanistic explanations (*p*s < 0.001).

**Table 2:** **Explanation coding in Experiment 1**.

**Explanation type**	**Biological organisms**	**Artifacts**
Mechanistic only	0.27	0.19
Functional only	0.52	0.76
Both	0.21	0.05
Neither	0.00	0.00

*The proportion of explanations of each type is indicated as a function of domain*.

#### Property generalizations

Patterns of generalization were analyzed by first averaging the three generalization ratings to the item with a shared function, creating a *function generalization score*, and averaging the three generalization ratings to the item with a shared mechanism, creating a *mechanism generalization score*. We then considered function generalization score and mechanism generalization score as two levels of the within-subjects variable *generalization score type*. Thus, generalization scores were analyzed in a mixed ANOVA with domain (2: biological organisms, artifacts) as a between-subjects factor and generalization score type (2: function generalization score, mechanism generalization score) as a within-subjects factor.

This analysis revealed a significant main effect of domain, *F*_(1, 250)_ = 37.81, *p* < 0.001, η^2^*_p_* = 0.13, with greater generalization overall to artifacts (*M* = 6.26, *SD* = 1.32) than to biological kinds (*M* = 5.33, *SD* = 1.06), as well as a significant interaction between domain and generalization score, *F*_(1, 250)_ = 5.58, *p* = 0.02, η^2^_*p*_ = 0.02. For biological organisms, a paired-samples *t*-test revealed greater generalization to items with shared functions (*M* = 5.61, *SD* = 1.66) than to those with shared mechanisms (*M* = 5.06, *SD* = 1.71), *t*_(125)_ = −2.33, *p* = 0.02. For artifacts, there was not a significant difference between generalization ratings to items with shared functions (*M* = 6.17, *SD* = 1.73) vs. those with shared mechanisms (*M* = 6.35, *SD* = 1.72), *t*_(125)_ = −0.90, *p* = 0.37.

Finally, to investigate whether there was competition between generalization on the basis of common mechanisms and generalization on the basis of common functions, we computed a correlation between mechanism and function generalization scores. The correlation was not significant, *r* = 0.046, *p* = 0.470, suggesting that finding one type of generalization plausible did not result in reduced plausibility for the alternative.

#### Property generalization as a function of explanation type

To test for the predicted relationships between explanation type and generalization (see Table 3), we conducted regression analyzes. First, we considered whether providing a functional explanation (0 = no, 1 = yes) predicted function generalization scores, with domain (1 = biological, 2 = artifact) and an interaction term as additional predictors. The interaction term was not significant (*p* = 0.68) and was dropped from the model. The resulting model revealed both functional explanation generation (*B* = 0.902, *SE* = 0.247, β = 0.223, *p* < 0.001) and domain (*B* = 0.487, *SE* = 0.209, β = 0.142, *p* = 0.021) as significant predictors, *R* = 0.277: participants were more likely to generalize properties to objects with shared functions if they generated functional explanations in response to the prompt, and when the objects were artifacts as opposed to biological organisms.

**Table 3:** **Generalization ratings in Experiment 1**.

**Mean ratings**	**Biological organisms**		**Artifacts**	
	**No (*N* = 35)**	**Yes (*N* = 91)**	**No (*N* = 24)**	**Yes (*N* = 102)**
**FUNCTIONAL EXPLANATION GENERATED?**				
Mechanism generalization score	5.62 (1.39)	4.82 (1.77)	6.26 (1.42)	6.37 (1.79)
Function generalization score	4.97 (1.54)	5.83 (1.66)	5.35 (1.57)	6.37 (1.72)
	**No (*N* = 65)**	**Yes (*N* = 61)**	**No (*N* = 96)**	**Yes (*N* = 30)**
**MECHANISTIC EXPLANATION GENERATED?**				
Mechanism generalization score	4.80 (1.78)	5.34 (1.60)	6.34 (1.81)	6.39 (1.43)
Function generalization score	5.99 (1.52)	5.19 (1.71)	6.39 (1.71)	5.49 (1.65)

*Average generalization ratings (on 1–9 scale) are indicated as a function of domain, with separate means for those participants who did and did not generate explanations of each type in response to the ambiguous explanation prompt. The means are followed in parentheses by standard deviations*.

We next considered whether providing a mechanistic explanation (0 = no, 1 = yes) predicted mechanism generalization scores, with domain (1 = biological, 2 = artifact) and an interaction term as additional predictors. Once again the interaction term was not significant (*p* = 0.148) and was dropped. The result was a model with domain as a significant predictor (*B* = 1.371, *SE* = 0.223, β = 0.375, *p* < 0.001), but not mechanistic explanation generation (*B* = 0.334, *SE* = 0.232, β = 0.088, *p* = 0.151), *R* = 0.363: participants were more likely to generalize properties to objects with shared mechanisms when the objects were artifacts as opposed to biological organisms, with no significant effect of whether they generated a mechanistic explanation in response to the prompt.

#### Distribution of responses

**Figure 1:**
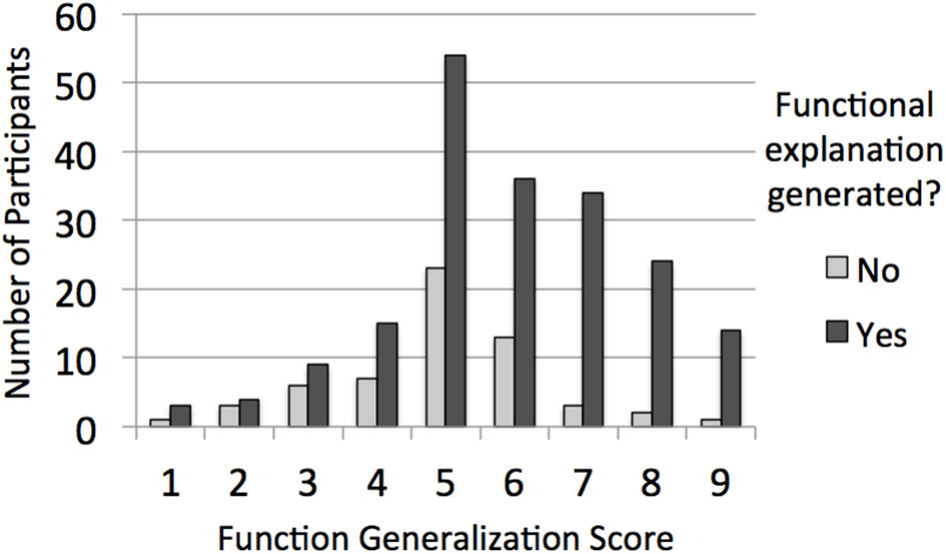
**For Experiment 1, the distribution of function generalization scores as a function of whether or not the participant generated a functional explanation in response to the ambiguous explanation prompt**.

To get a better sense for how having generated a functional explanation related to the distribution of function generalization scores across participants (i.e., the predicted result for which we found support), we additionally classified participants into nine groups based on their function generalization score, effectively truncating the score to one digit (so, for instance, scores greater than or equal to 1, but less than 2, were coded as “1”). Figure 1 plots the resulting distribution as a function of whether or not participants generated a functional explanation in response to the prompt, and suggests that the effect of explanation type on generalization was not restricted to a small subset of participants. Because we did not find an interaction between having generated a functional explanation and domain when it came to predicting function generalization scores, we collapsed across domain.

#### Summary of findings

In sum, Experiment 1 partially confirmed our key predictions: generating a functional explanation predicted the extent to which participants generalized properties to items with shared functions, and this relationship did not differ across domains. However, we did not find a significant relationship between having generated a mechanistic explanation and generalizing to items with shared mechanisms. We also found no evidence for competition between generalization on the basis of shared mechanisms and of shared functions.

Experiment 1 additionally found that participants had a higher baseline tendency to provide functional explanations than mechanistic explanations across both domains, with a matching tendency to generalize more to items with shared functions over shared mechanisms, but only for biological organisms. The experiment also revealed greater generalization overall to artifacts than to biological organisms. We return to these findings in the general discussion.

## Experiment 2: prompted explanations influence patterns of generalization

While the findings from Experiment 1 support the hypothesis that explanation guides generalization, the correlational design of the experiment prevents causal conclusions from being drawn. It could be that explanation type influenced generalization, but it is also possible that intuitions about generalization influenced explanation, or that both explanations and generalizations had a common cause. Experiment 2 aims to examine the causal relationship between explanation type and generalization by experimentally manipulating the kind of explanation a participant considers and soliciting subsequent generalization judgments. If the way a property is explained has a causal impact on how it is generalized, then relative to participants experimentally induced to consider mechanistic explanations, participants experimentally induced to consider functional explanations should be more likely to generalize properties on the basis of shared functions and less likely to generalize properties on the basis of shared mechanisms, mirroring and extending the findings from Experiment 1.

Experiment 2 induces participants to consider a particular kind of explanation in two ways. In the initial “priming phase” of the experiment, participants receive the same mechanistic and functional information about a novel category as that provided in Experiment 1, but one explanation is subsequently privileged [e.g., “So now you know why narps have a speckled pattern: narps have a speckled pattern because of the XP2 gene (to attract butterflies)”]. Generalization judgments are then solicited for the categories introduced in the priming phase to investigate whether experimentally privileging a particular explanation has the predicted effect on generalization.

The priming phase is followed by a *generation* phase in which participants learn about two novel categories and are prompted to generate their own explanations for their properties. Although the categories in the generation phase are distinct from those in the priming phase, this design allows us to investigate whether the priming phase effectively induces or “primes” a particular explanation type, leading to explanations and generalizations that are more consistent with that type in the generation phase. By varying whether the two categories in the priming phase and in the generation phase come from the same domain or from different domains, we can additionally investigate whether explanatory modes are proprietary to domains—that is, for example, whether the kind of “functional reasoning” that might occur for artifacts is related to that which occurs for biological organisms.

### Methods for experiment 2

#### Participants

Four-hundred-eighty-four workers on Amazon Mechanical Turk (270 male, 210 female, 4 other/unspecified; Age: *M* = 32, *SD* = 10) completed the task in exchange for monetary compensation. All participants provided informed consent, following a human subjects protocol approved by UC Berkeley's Institutional Review Board. Participation was restricted to workers with IP addresses within the United States, a HIT approval ratings of 95% or higher, and at least 50 previously-approved HITs. An additional 224 participants were excluded from analyzes due to an experimenter error in the stimulus materials (49), for leaving numerical responses or explanations blank (128), or for failing an instructional manipulation check (47).

#### Materials and procedure

The stimuli from Experiment 1 were used in Experiment 2, with the following changes to the procedure. Instead of seeing a single category, each participant was presented with four. For the first two categories presented in the “priming phase,” explanations were provided rather than prompted. The answers were both mechanistic for participants in the *mechanistic prime* condition, and both functional for participants in the *functional prime* condition. For example:

A narp is a kind of plant with a speckled pattern. Biologists have discovered that in narps, the speckled pattern is caused by the XP2 gene. Having a speckled pattern attracts butterflies, which play a role in pollination.

[*mechanistic prime*] So now you know why narps have a speckled pattern: narps have a speckled pattern because of the XP2 gene.

[*functional prime*] So now you know why narps have a speckled pattern: narps have a speckled pattern to attract butterflies.

After each category in the priming phase, novel items were introduced and generalization judgments were solicited as in Experiment 1.

For the next two categories in the “generation phase,” participants provided explanations in response to an ambiguous why-question, as in Experiment 1. For instance, participants who learned about narps in the priming phase might be asked about brolligs in the generation phase:

A brollig is a kind of reptile with stripes. In brolligs, the stripes are caused by minerals in the reptile's diet. Having stripes also has an important function: it helps brolligs hide from predators.

In a sentence, why do brolligs have stripes?

After each category in the generation phase, novel items were introduced and generalization judgments were solicited as in Experiment 1.

The two categories in the priming phase were always from the same domain (both biological organisms or artifacts), and the subsequent two categories in the generation phase were always from the same domain (both biological organisms or artifacts). However, the domain could either remain the same or change across the two phases–it matched for about half of the participants and did not match for the remainder.

The four categories in each domain were divided into two sets such that participants either received set A or set B. There were therefore 16 conditions total to which participants were randomly assigned: 2 (mechanistic or functional prime) × 2 (domain of provided explanations: biological organisms or artifacts) × 2 (domain of prompted explanations: biological organisms or artifacts) × 2 (category set: A or B).

### Results and discussion for experiment 2

We first report the data from the first two sets of judgments in the priming phase (for which explanations were provided), before considering its impact on explanations and generalizations for the novel categories in the generation phase (for which explanations were prompted from participants).

#### Generalization ratings in priming phase

Mechanism and function generalization scores were computed as in Experiment 1, with the scores averaged across the two target categories to create a single pair of scores for the priming phase (see **Table 5**). These scores were analyzed as two levels of a within-subjects factor (generalization score type: function generalization score, mechanism generalization score) in a mixed ANOVA with priming explanation type (2: mechanistic, functional) and priming explanation domain (2: biological organisms, artifacts) as between-subjects factors.

**Figure 2:**
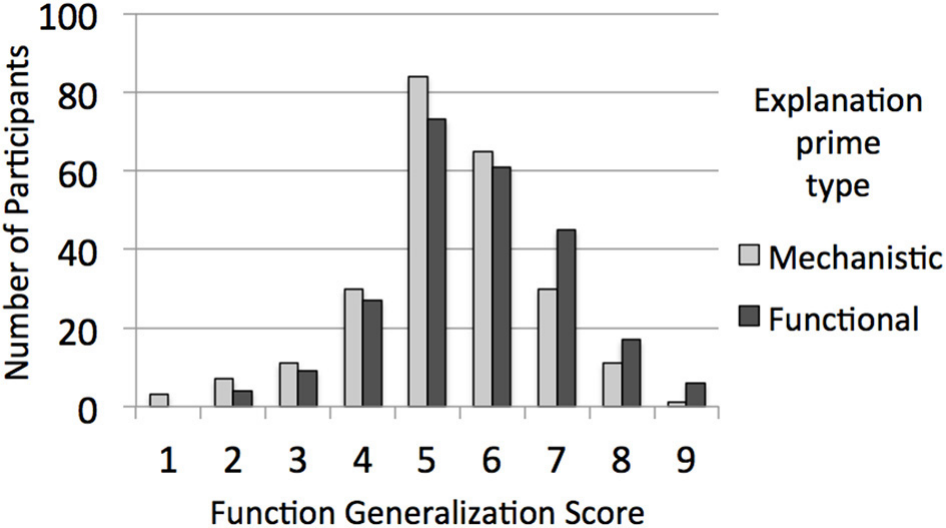
**For Experiment 2, the distribution of function generalization scores from the priming phase as a function of whether the participant was provided with functional explanations or mechanistic explanations**.

This analysis revealed the predicted interaction between generalization score type and priming explanation type, *F*_(1, 480)_ = 9.78, *p* = 0.002, η^2^_*p*_ = 0.02. Participants generalized properties more to items with shared functions when functional explanations were primed (*M* = 6.07, *SD* = 1.36) than when mechanistic explanations were primed (*M* = 5.69, *SD* = 1.37), *t*_(482)_ = −3.054, *p* = 0.002, *r* = 0.14. There was not a significant difference in generalization to properties with shared mechanisms in response to priming explanation type (functional: *M* = 5.57, *SD* = 1.46; mechanistic: *M* = 5.67, *SD* = 1.37), *p* = 0.44. These findings mirror those for Experiment 1, with an impact of explanation type on function but not mechanism generalization scores, but for experimentally provided rather than freely chosen explanation types. To visually represent this result, Figure 2 plots the distribution of function generalization scores (discretized as in Experiment 1) as a function of experimental condition. Cumulative density functions (CDFs) were also plotted for each condition, and visual inspection suggested a uniform shift consistent with a difference in means for the whole distribution and not only for a subsample.

There were several additional significant effects of the mixed ANOVA: a main effect of generalization score type, *F*_(1, 480)_ = 12.026, *p* = 0.001, η^2^_*p*_ = 0.024, a main effect of domain, *F*_(1, 480)_ = 82.014, *p* < 0.001, η^2^_*p*_ = 0.146, and an interaction between these, *F*_(1, 480)_ = 19.874, *p* < 0.001, η^2^_*p*_ = 0.040. Overall, there was greater property generalization to artifacts than to biological organisms, and to items with a shared function over items with a shared mechanism, but this difference was greater for biological organisms than for artifacts (see **Table 5**). These findings again mirror those from Experiment 1, where there was greater generalization to artifacts than to biological organisms, and greater generalization to items with shared functions than to items with shared mechanisms, but only significantly so for biological organisms.

Finally, to investigate whether there was competition between generalization on the basis of common mechanisms and generalization on the basis of common functions, we computed a correlation between mechanism and function generalization scores as in Experiment 1. The correlation was significant, *r* = 0.210, *p* < 0.001, but positive. That is, generalization on one basis was associated with *greater* generalization on the other, again contrary to the idea that mechanistic and functional bases for generalization reflect competing modes of reasoning.

#### Explanations generated in generation phase

To analyze the effects of the explanation primes on the two prompted explanations, all explanations were coded as in Experiment 1 (see Table 4). An initial coder coded all explanations blind to condition; a second coder coded 25% blind to condition, yielding 99.5% agreement, with the single disagreement resolved in favor of the first coder. With two prompted explanations, each participant could have a *mechanistic explanation score* between 0 and 2 and a *functional explanation score* between 0 and 2.

**Table 4:** **Explanation coding in Experiment 2**.

**Explanation type**	**Mechanism prime**		**Function prime**	
	**Biological organisms prompted**	**Artifacts prompted**	**Biological organisms prompted**	**Artifacts prompted**
Mechanistic only	0.42	0.28	0.28	0.21
Functional only	0.41	0.60	0.58	0.72
Both	0.17	0.10	0.13	0.07
Neither	0.00	0.00	0.00	0.00

*The proportions of explanations of each type are indicated as a function of explanation type in the priming phase and the domain of prompted explanations in the generation phase*.

Mechanistic explanation score was analyzed as the dependent variable in an ANOVA with priming explanation type (2: mechanistic, functional), prompted explanation domain (2: biological organisms, artifacts), and whether the priming explanation matched the prompted explanations in domain (2: same domain, different domain) as independent variables. This analysis revealed a main effect of priming explanation type, *F*_(1, 476)_ = 16.43, *p* < 0.001, η^2^_*p*_ = 0.03, and a main effect of prompted explanation domain, *F*_(1, 476)_ = 13.68, *p* < 0.001, η^2^_*p*_ = 0.03. Participants were more likely to provide mechanistic explanations when the explanation prime was mechanistic (*M* = 1.00, *SD* = 0.90) than when it was functional (*M* = 0.68, *SD* = 0.86), and when the prompted explanations concerned biological organisms (*M* = 0.99, *SD* = 0.93) than when they concerned artifacts (*M* = 0.70, *SD* = 0.83). Notably, there was not a significant interaction between priming explanation type and domain match (*p* = 0.12), suggesting that the effect of priming explanation type was not stronger for same-domain than cross-domain cases.

An equivalent analysis with functional explanation score as the dependent variable revealed a single significant main effect of priming explanation type, *F*_(1, 476)_ = 13.58, *p* < 0.001, η^2^_*p*_ = 0.03. Participants were more likely to provide functional explanations when the explanation prime was functional (*M* = 1.51, *SD* = 0.77) than when it was mechanistic (*M* = 1.23, *SD* = 0.89). Again, the interaction between priming explanation type and domain match was not significant (*p* = 0.37).

As in Experiment 1, participants were more likely to provide functional explanations than mechanistic explanations in both domains (*p*s < 0.01).

#### Generalization ratings in generation phase

Participants' generalization ratings for the final two categories–for which explanations were prompted–were combined into a pair of *mechanism* and *function generalization scores* for analysis of generalizations in the generation phase, as they were for generalizations in the priming phase (see Table 5).

**Table 5:** **Generalization ratings in Experiment 2**.

**Mean ratings**	**Priming phase**				**Generation phase**			
	**Mechanistic prime**		**Functional prime**		**Mechanistic prime**		**Functional prime**	
	**Bio**	**Art**	**Bio**	**Art**	**Bio**	**Art**	**Bio**	**Art**
Mechanism generalization score	5.11 (1.35)	6.22 (1.16)	4.93 (1.37)	6.17 (1.28)	5.13 (1.37)	6.26 (1.20)	5.06 (1.49)	5.85 (1.17)
Function generalization score	5.45 (1.24)	5.93 (1.45)	5.82 (1.32)	6.30 (1.36)	5.83 (1.32)	5.88 (1.11)	5.97 (1.41)	5.96 (1.27)

*Average generalization ratings (on 1–9 scale) are indicated as a function of explanation condition and domain, with different scores for the priming phase (explanations provided) and the generation phase (explanations prompted). Note that scores for the generation phase are indicated as a function of prime type (an experimental manipulation), not as a function of the explanation type participants generated themselves. The means are followed in parentheses by standard deviations*.

**Figure 3:**
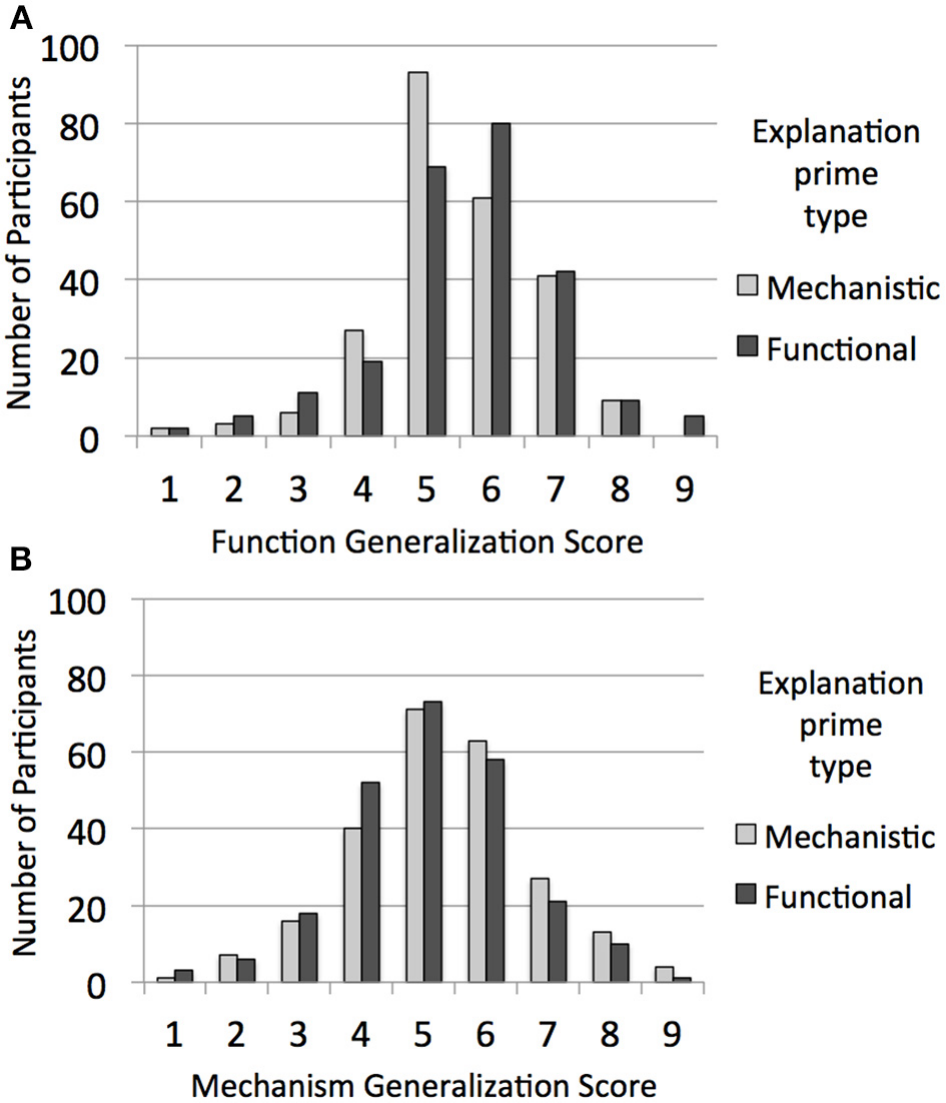
**For Experiment 2, the distribution of (A) function generalization scores and (B) mechanism generalization scores from the generation phase as a function of whether the participant was provided with functional explanations or mechanistic explanations in the priming phase**.

Generalization score type was a within-subjects factor in a mixed ANOVA with primed explanation type (2: mechanistic prime, functional prime), domain of prompted explanations (2: biological organisms, artifacts), and whether the priming explanations matched the prompted explanations in domain (2: same domain, different domain) as independent variables. This analysis revealed the predicted interaction between generalization score type and primed explanation type, *F*_(1, 476)_ = 4.29, *p* = 0.04, η^2^_*p*_ = 0.01. Participants generalized marginally more to items with shared mechanisms when mechanistic explanations were primed (*M* = 5.71, *SD* = 1.41) than when functional explanations were primed (*M* = 5.46, *SD* = 1.39), *p* = 0.05, with the opposite (non-significant) pattern of generalization to items with shared functions when functional explanations were primed (*M* = 5.97, *SD* = 1.34) vs. mechanistic explanations (*M* = 5.85, *SD* = 1.22), *p* = 0.32 (see Figure 3).

There were several additional significant effects. There was a main effect of generalization score type, *F*_(1, 476)_ = 15.79, *p* < 0.001, η^2^_*p*_ = 0.03, with greater generalization to items with shared functions (*M* = 5.97, *SD* = 1.34) than to those with shared mechanisms (*M* = 5.46, *SD* = 1.39). There was also a main effect of prompted explanation domain, *F*_(1, 476)_ = 36.39, *p* < 0.001, η^2^_*p*_ = 0.07, which was qualified by interactions with generalization score, *F*_(1, 476)_ = 31.06, *p* < 0.001, η^2^_*p*_ = 0.06, with domain match, *F*_(1, 476)_ = 6.52, *p* = 0.01, η^2^_*p*_ = 0.01, and with both of these in a three-way interaction, *F*_(1, 476)_ = 6.13, *p* = 0.01, η^2^_*p*_ = 0.01. While generalization to items with a shared function was not influenced by prompted explanation domain or by domain match (*p*s > 0.80), generalization to items with a shared mechanism was greater for artifacts (*M* = 6.06, *SD* = 1.20) than for biological organisms (*M* = 5.10, *SD* = 1.43), *F*_(1, 480)_ = 66.25, *p* < 0.001, η^2^_*p*_ = 0.12, with a larger difference when the domain of the priming explanations matched that of the prompted explanations, *F*_(1, 480)_ = 12.24, *p* = 0.001, η^2^_*p*_ = 0.03. Notably, there was not a significant interaction between priming explanation type and domain match, *p* = 0.15, suggesting that the effect of the explanation type prime was not detectably stronger for same-domain than cross-domain cases.

Finally, we computed a correlation between mechanism and function generalization scores. The correlation was not significant, *r* = −0.042, *p* = 0.362.

#### Summary of key findings

In sum, Experiment 2 partially confirmed our key prediction that an experimentally privileged explanation type would influence subsequent generalization. The findings for the priming phase mirrored those for Experiment 1, with explanation type successfully predicting generalization to items with shared functions, but not to items with shared mechanisms. The findings for the generation phase revealed the predicted interaction between explanation prime type and generalization type, with weak but directionally-appropriate effects for generalizations to both items with shared functions and to those with shared mechanisms.

Experiment 2 went beyond Experiment 1 in establishing a *causal* relationship between explanation and generalization, but also in demonstrating the potential to prime an explanatory mode. Priming mechanistic explanations increased the proportion of mechanistic explanations subsequently provided, and priming functional explanations increased the proportion of functional explanations subsequently provided. The impact of prime type also extended to generalization judgments, and there was no evidence for stronger effects of priming within-domain than across-domain, suggesting some psychological reality to explanatory modes that are not proprietary to domains. However, given that the absence of a stronger effect for same-domain cases is a null result, it would be especially worthwhile to revisit this question with larger samples and more sensitive measures. It is also worth acknowledging more general difficulties in drawing conclusions from null results.

Finally, Experiment 2 also replicated Experiment 1 in finding no evidence that “mechanistic generalization” is in competition with “functional generalization,” and in documenting a higher baseline tendency to generate functional explanations in both domains and to generate mechanistic explanations for biological organisms more often than for artifacts. Both experiments also revealed common trends in generalization, with a greater baseline tendency to generalize to items with shared functions over shared mechanisms (especially for biological organisms), and to generalize properties more to artifacts than to biological organisms.

## General discussion

Our findings suggest a relationship between how a property is explained and how it is generalized, whether the explanation is generated freely (Experiment 1), provided experimentally (Experiment 2, priming phase), or induced experimentally (Experiment 2, generation phase). Across experiments, we find that functional explanations reliably influence the extent to which properties are generalized on the basis of shared functions, with weaker evidence of a comparable relationship between mechanistic explanations and generalizations on the basis of shared mechanisms. We also find that an explanatory mode can be experimentally induced by providing examples of that explanation type, and that this induction influences subsequent generalizations, both within and across domains. These findings suggest that mechanistic and functional explanations reflect explanatory schemata that are abstracted beyond individual domains, and that these schemata play a role in how we generalize from the known to the unknown. Our findings also suggest that “reasoning mechanistically” (as reflected by generalization on the basis of common parts and processes) does not seem to preclude “reasoning functionally” (as reflected by generalization on the basis of common functions), and vice versa.

The finding that mechanistic and functional explanations guide property generalization contributes to existing work demonstrating a relationship between explanation and generalization (Sloman, 1994; Rehder, 2006; Vasilyeva and Coley, 2013). Unlike previous work, however, the current experiments considered cases for which participants received causal information that supported multiple explanations. In such cases, differential patterns of generalization cannot be accounted for by appeal to differences in the causal relationships that a reasoner knows or learns, as this was matched across participants. Instead, the findings suggest that generalizations can be guided by the particular explanation a reasoner privileges, whether that explanation is privileged spontaneously or through experimental prompting.

We found a baseline preference for generating functional explanations over mechanistic explanations, and also for generalizing on the basis of shared functions over shared mechanisms. These findings are consistent with the idea that teleo-functional reasoning is a cognitive default (Kelemen, 1999; Kelemen and Rosset, 2009), or with the idea that people defeasibly prefer functional explanation when there's a good fit between an object's structure and its function (Lombrozo et al., 2007). However, this could also be a consequence of our stimulus materials rather than a widespread feature of human cognition. For example, it's possible that because our mechanistic explanations often involved fictional genes or compounds, participants felt that they understood the functional explanations better, and this difference in understanding generated a difference in explanatory preferences and in generalization. While it's not possible to generate explanations that are identical in all respects except for being functional or mechanistic, it would nonetheless be worthwhile to pursue experiments along the lines of those presented here with explanations that have been matched in terms of familiarity, plausibility, and other dimensions.

Our experiments suggest a more reliable relationship between functional explanations and “functional generalization” than between mechanistic explanations and “mechanistic generalization.” We did not anticipate this difference, but there are a few plausible ways in which it might be explained. First, the difference could result from the potential difference in understanding already noted: it could be that the functional explanations generated greater understanding, and that depth of understanding facilitated generalization. Second, it's also the case that proximate parts and processes typically support mechanistic explanations, whereas causal consequences don't always support functional explanations. That is, proximate parts and processes almost always contribute to a mechanistic explanation for some property, even if they occasionally play a secondary role as enabling conditions or other subsidiary causes. In contrast, features can have functional consequences that do not support functional explanations at all: noses hold up glasses, but we cannot explain noses by saying they are “for holding glasses” (Lombrozo and Carey, 2006). As a result, the provision of a mechanistic explanation could have been essentially redundant with the mechanistic information provided, while the provision of a functional explanation was more diagnostic of how the reasoner was actually representing the explanatory role of the functional information. Whether either of these ideas hold up to further scrutiny, the asymmetry between functional and mechanistic explanations found in our studies is useful in ruling out an alternative explanation for the results: that they were merely the result of the demand characteristics of the tasks. Were this the case, one would expect parallel effects for both explanation types.

These experiments are among the first to explore the role of domain in property generalization, although the results with respect to domain are somewhat mixed. Both experiments found greater generalization overall to artifacts than to biological organisms, and this pattern was partially matched by domain differences in explanation: functional explanations were produced more frequently overall for both domains (matching the greater generalization to items with shared functions), but mechanistic explanations were produced more frequently for biological organisms than for artifacts, a finding that was not matched by a corresponding trend in generalization. These results are broadly consistent with prior work suggesting a privileged status for functional explanations in biological domains (e.g., Atran, 1995; Keil, 1995; Heussen, 2010). However, we hesitate to draw strong conclusions about entire domains on the basis of a handful of items. Instead, we think the most important findings concerning domain are that the influence of explanation on generalization was robust across biological organisms and artifacts, and that explanatory modes can be induced both within and across domains.

In concluding, we suggest that generating and evaluating explanations is an important mechanism by which prior beliefs are consulted and brought to bear on a given task. While explanation is surely not the only process that invokes prior beliefs, it appears to be a particularly powerful one (Lombrozo, 2006; Williams and Lombrozo, 2013; see also Vasilyeva and Coley, 2013). Research in education, for example, suggests that prompting learners to generate explanations–even to themselves–facilitates the integration of the material being explained with prior beliefs (Chi et al., 1994). Some approaches to learning and generalization within artificial intelligence also rely on an “explanation” as a way to invoke relevant domain-knowledge (e.g., DeJong, 2004; see also Ahn et al., 1992). Understanding the role of explanation in generalization will require–among other things–an understanding of which prior beliefs particular explanations invoke (e.g., Chin-Parker and Bradner, 2010; Landy and Hummel, 2010), why those particular beliefs are invoked, and how those beliefs constrain generalization.

The distinction between mechanistic and functional modes of explanation may shed some light on these processes. Explanatory modes or “stances” are typically posited to explain systematic patterns of explanation and generalization. While the details vary, mushrooms, computers, and kangaroos can all be reasoned about “mechanistically,” in terms of causal parts and processes, or “functionally,” in terms of functions and goals. Each mode may involve a characteristic kind of representation or inference that makes it useful for particular situations. For example, Lombrozo (2010) suggests that different aspects of causal structure are privileged depending on whether a system is construed mechanistically or functionally. Explanatory modes can thus be conceptualized as sets of higher-order generalizations that constrain reasoning. Depending on which mode is adopted, different aspects of prior beliefs will be invoked, different kinds of novel beliefs will be generated, and subsequent patterns of generalization will be influenced correspondingly. Characterizing the nature of different kinds of explanations can thus provide key insights into the nature of inductive constraints, and the processes by which prior beliefs guide inference.

### Conflict of interest statement

The authors declare that the research was conducted in the absence of any commercial or financial relationships that could be construed as a potential conflict of interest.
